# Development and validation of multi-omic prognostic signature of anoikis-related genes in liver hepatocellular carcinoma

**DOI:** 10.1097/MD.0000000000036190

**Published:** 2023-11-17

**Authors:** Dongxiao Ding,, Dianqian Wang,, Yunsheng Qin

**Affiliations:** a Department of Thoracic Surgery, The People’s Hospital of Beilun District, Ningbo, Zhejiang, China; b Health Science Center, Ningbo University, Zhejiang, China; c Department of Hepatological Surgery, First Affiliated Hospital of Medical School of Zhejiang University, Hangzhou, Zhejiang, China.

**Keywords:** bioinformatics, gene signature, immune microenvironment, liver hepatocellular carcinoma, prognosis

## Abstract

Liver hepatocellular carcinoma (LIHC) is characterized by high morbidity, rapid progression and early metastasis. Although many efforts have been made to improve the prognosis of LIHC, the situation is still dismal. Inability to initiate anoikis process is closely associated with cancer proliferation and metastasis, affecting patients’ prognosis. In this study, a corresponding gene signature was constructed to comprehensively assess the prognostic value of anoikis-related genes (ARGs) in LIHC. Using TCGA-LIHC dataset, the mRNA levels of the differentially expressed ARGs in LIHC and normal tissues were compared by Student *t* test. And prognostic ARGs were identified through Cox regression analysis. Prognostic signature was established and then externally verified by ICGC-LIRI-JP dataset and GES14520 dataset via LASSO Cox regression model. Potential functions and mechanisms of ARGs in LIHC were evaluated by functional enrichment analyses. And the immune infiltration status in prognostic signature was analyzed by ESTIMATE algorithm and ssGSEA algorithm. Furthermore, ARGs expression in LIHC tissues was validated via qRT-PCR and IHC staining from the HPA website. A total of 97 differentially expressed ARGs were detected in LIHC tissues. Functional enrichment analysis revealed these genes were mainly involved in MAP kinase activity, apoptotic signaling pathway, anoikis and PI3K-Akt signaling pathway. Afterward, the prognostic signature consisting of BSG, ETV4, EZH2, NQO1, PLK1, PBK, and SPP1 had a moderate to high predictive accuracy and served as an independent prognostic indicator for LIHC. The prognostic signature was also applicable to patients with distinct clinical parameters in subgroup survival analysis. And it could reflect the specific immune microenvironment in LIHC, which indicated high-risk group tended to profit from ICI treatment. Moreover, qRT-PCR and IHC staining showed increasing expression of BSG, ETV4, EZH2, NQO1, PLK1, PBK and SPP1in LIHC tissues, which were consistent to the results from TCGA database. The current study developed a novel prognostic signature comprising of 7 ARGs, which could stratify the risk and effectively predict the prognosis of LIHC patients. Furthermore, it also offered a potential indicator for immunotherapy of LIHC.

## 1. Introduction

As the most common subtype of primary liver cancer, liver hepatocellular carcinoma (LIHC) is the second deadliest cancer globally.^[1]^ In recent years, global incidence and death rate of LIHC presents a gradual upward trend. Scientists predict LIHC is projected to cause more than one million cancer-related deaths in 2025.^[2]^ Currently, although the risk factors for LIHC occurrence, such as Hepatitis B virus infection, aflatoxin B1 exposure and liver cirrhosis, have been well identified and comprehensive treatment strategies, including surgery, chemotherapy, targeted drug treatment, immunotherapy and liver transplantation, have been widely used in clinical application, dismal 5-year survival rate of <20% and early diagnosis rate of <30% still exist in some developing countries.^[3,4]^ High treatment expenditure, severe drug adverse reaction and lack of advanced diagnosis and treatment technology also reduce patients’ treatment participation.^[5]^ In all, survival status of LIHC patients is unsatisfactory. In addition, with the in-depth study of tumor heterogeneity, traditional prognostic assessment according to TNM stage alone is being challenging. Thus, developing a prognostic signature based on molecular profiles of LIHC is promising.

Anoikis is a specific form of apoptotic event that can be initiated when cells lose their interaction with the adjacent extracellular matrix (ECM).^[6,7]^ It is indispensable to maintain the stability of body tissues, and its main function is to prevent abnormal cell growth or cell adhesion to abnormal ECM. For example, when cells are detached from ECM, the loss of integrin-mediated survival signals will activate Bcl-2-modifying factor, thus activating the process of anoikis.^[8]^ Nevertheless, cancer cells can develop anoikis resistance occasionally through circumventing death signaling pathway. These cells with anoikis resistance may survival while spreading from the primary site to distant organs and participate in the emergence of metastases.^[6,9]^ In this process, anoikis-resistant cancer cells also show high VEGFA expression and promote angiogenesis.^[10]^ At present, dysregulation of cell apoptosis is considered as a key factor in tumorigenesis and tumor development. Thus, exploring expression status and prognostic value of anoikis-related genes (ARGs) in LIHC as well as their correlations with tumor microenvironment is of great significance.

Previous studies have reported that ARGs participated in the process of tumor metastasis and progression and was associated with patients’ prognosis in various human tumor types. For example, Takagi et al suggested high KLF5 expression contributed to anoikis resistance and poor prognosis in colorectal cancer patients. In addition, through promoting cell proliferation and cancer stem cell-like properties, KLF5 also contributed to hepatic metastases of colorectal cancer.^[11]^ Shimokawa et al reported NQO1 activation was associated with LIHC metastasis and poor prognosis through generating anoikis resistance in anchorage-independent culture.^[12]^ Kim et al suggested that CEACAM6 overexpression was correlated with unfavorable prognosis in lung adenocarcinoma and inhibited anoikis via the activation of Src-FAK signaling pathway.^[13]^ In pancreatic cancer, Jiang et al suggested upregulated EDIL3 expression was related to shorter survival time, and inhibition of EDIL3 disrupted anoikis resistance and anchorage-independent tumor growth.^[14]^

The abovementioned articles have proved the prognostic value of ARGs in corresponding cancer types. As for LIHC, some studies have started to focus on the role of ARGs in LIHC, but few studies discussed the relationship between integrated ARGs and survival outcomes. In this study, we analyzed the expression status, mutation status and prognostic performance of ARGs in LIHC. We built up and verified an ARGs-related prognostic signature using integrated expression data and survival data. Besides, the correlation between tumor immune microenvironment and the signature was also evaluated. The current study may offer powerful evidences on risk stratification, prognosis evaluation and immunotherapy of LIHC.

## 2. Materials and methods

### 2.1. Data processing

On June 18, 2022, RNA-sequencing data and corresponding clinical data of 374 LIHC samples and 50 normal liver samples were downloaded from The Cancer Genome Atlas (TCGA, https://portal.gdc.cancer.gov/) database. Standardized copy number variation (CNV) and single nucleotide variation (SNV) data of these samples were acquired from UCSC Xena (https://xena.ucsc.edu/). Setting a relevance score > 0.5 in GeneCards (https://www.genecards.org/), a total of 404 anoikis-related genes (ARGs) were obtained. The gene list was shown in Supplementary table 1, http://links.lww.com/MD/K768. Statistical analyses were conducted in R software V4.0.2.

### 2.2. Expression analysis and mutation analysis

Using “edgeR” package in R, the differential expression analysis of all the genes in TCGA-LIHC dataset was conducted with the following threshold: false discovery rate (FDR) < 0.05 and |fold change (FC)| > 1. Afterward, differentially expressed ARGs (DEARGs) were acquired by interacting with the 404 ARGs. The process was visualized by Venn plot. The expression data was standardized to fragments per kilobase million values and was compared by Student *t* test. Using “reshape2” and “limma” packages, the box plots showed the transcriptional levels of DEARGs in LIHC tissues and normal liver tissues. CNV and SNV status of target genes in LIHC as well as the waterfall plot were analyzed and visualized by “maftools” package. The corresponding variation locations of these genes on chromosomes were visualized by “RCircos” package.

### 2.3. Protein-protein interaction network construction and functional enrichment analysis

The abovementioned expression analysis identified the DEARGs in LIHC. Next, based on STRING (https://cn.string-db.org/) website,^[15]^ the associations among these genes were expressed as a protein-protein interation (PPI) network. To investigate the potential molecular mechanisms and biological functions of DEARGs, gene ontology (GO) analysis and Kyoto Encyclopedia of Genes and Genomes (KEGG) pathway analysis were performed based on “clusterProfiler” package. GO analysis included 3 dimensions: biological process (BP), cellular component (CC) and molecular function (MF). The threshold of functional enrichment analysis was set as FDR < 0.05 and q-value < 0.2.

### 2.4. Prognostic analysis and prognostic signature construction

Setting the median expression level as cutoff, the prognostic performance of DEARGs was also analyzed by “survival” package in R and was visualized as a forest graph. After prognostic-related DEARGs were screened out, LASSO cox regression analysis in “glmnet” package was used to establish the prognostic signature. $Riskscore=\underset{i=1}{\overset{n}{\sum }}\;\left(Expi\;\times Coei\right)$. *Expi* was the expression value of each prognostic-related DEARG in LIHC, and the *Coei* was the corresponding multivariable Cox regression coefficient. Selecting the median risk score as cutoff, patients were assigned to high-risk and low-risk group. Overall survival curves of the 2 groups were compared by Kaplan–Meier analysis in “survival” package. Predictive accuracy of the prognostic signature was evaluated by Time ROC analysis in “timeROC” package. ICGC-LIRI-JP dataset and GSE14520 dataset were also obtained from International Cancer Genome Consortium (ICGC, https://dcc.icgc.org/) and Gene Expression Omnibus (GEO, https://www.ncbi.nlm.nih.gov/geo/) database to externally validate predictive power, respectively. Moreover, univariate and multivariate Cox regression analysis were conducted to explore the independent prognosis indicators. Afterward, subgroup survival analyses were utilized to explore whether the prognostic signature was also applicable to patients with different clinical parameters. In addition, the risk score differences in multiple subgroups was also evaluated.

### 2.5. Immune analysis of the prognostic signature

Previous studies suggested immune microenvironment was of great importance in the occurrence and progression of tumor. To evaluate the overall immune microenvironment in the 2 groups, the ESTIMATE algorithm was used to assess the StromalScore, EstimateScore, and ImmuneScore.^[16]^ Next, the infiltrating levels of 24 distinct immunocyte subsets in high-risk and low-risk group were also investigated by ssGSEA algorithm.^[17]^ Furthermore, the association between risk score and some common immune checkpoints (CD274, CTLA4, HAVCR2, TIGIT, PDCD1, LAG3, PDCD1LG2, and SIGLEC15) expression was also analyzed.

### 2.6. Validation of expression status of the risk score-related ARGs

Approved by the Ethics Committee of the Second Affiliated Hospital of Nanchang University, tumorous tissues and adjacent normal tissues from 40 LIHC patients without any preoperative therapy were collected for the present study. All these patients were informed and provided written consent. Quantitative real-time polymerase chain reaction (qRT-PCR) was performed as previously described.^[18]^ The 2^−ΔΔCt^ method was used to calculate the expression of each gene relative to the housekeeping gene. Primers used in this study were presented in Supplementary table 2, http://links.lww.com/MD/K769. The transcriptional level of risk score-related ARGs in tumorous tissues and adjacent normal tissues were compared by Student *t* test. Furthermore, we also downloaded the immunohistochemical (IHC) staining data from The Human Protein Atlas (HPA, https://www.proteinatlas.org/) to verify their protein levels.^[19]^

## 3. Results

### 3.1. Identification of prognostic-related DEARGs

**Figure 1.**
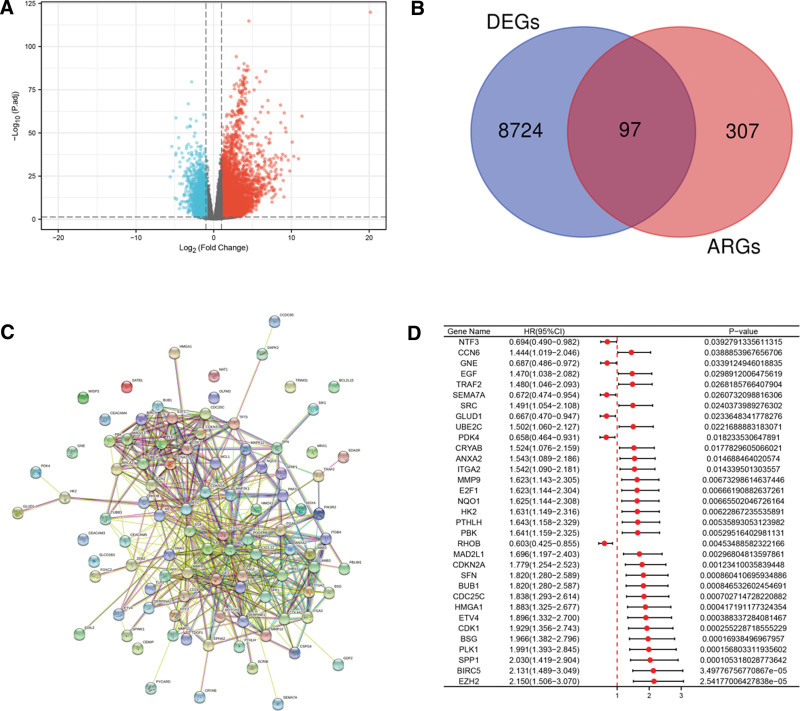
Identification of prognostic-related ARGs. (A) Volcano map showed the all differentially expressed genes in TCGA-LIHC dataset. The red points represented the upregulated genes, the blue points represented the downregulated genes, and the gray points represented no statistically differentially expressed genes with the cutoff of FDR < 0.05 and |fold change (FC)| > 1. (B) Venn plot of ARGs and DEGs. Ninety-seven ARGs were identified as DEARGs (C) PPI network of the 97 DEARGs. (D) The prognostic performance of the 33 prognostic-related ARGs in LIHC. Patients with low NTF3, GNE, SEMA7A, GLUD1, PDK4 and RHOB expression were related to a worse prognosis, while LIHC patients with high CCN6, EGF, TRAF2, SRC, UBE2C, CRYAB, ANXA2, ITGA2, MMP9, E2F1, NQO1, HK2, PTHLH, PBK, MAD2L1, CDKN2A, SFN, BUB1, CDC25C, HMGA1, ETV4, CDK1, BSG, PLK1, SPP1, BIRC5 and EZH2 expression showed a worse prognosis. ARG = anoikis-related gene, DEARG = differentially expressed anoikis-related gene, FDR = false discovery rate, LIHC = liver hepatocellular carcinoma.

Setting the FDR < 0.05 and |fold change (FC)| > 1, expression status of all genes in TCGA-LIHC dataset (374 LIHC samples and 50 normal liver samples) was analyzed. In total, 8821 genes were upregulated or downregulated in LIHC tissues (Fig. 1A). Next, compared with the 404 ARGs from GeneCards, the intersection showed 97 ARGs were identified as DEARGs, which included 67 upregulated genes and 30 downregulated genes (Fig. 1B). To better visualize the 97 DEARGs and their potential interactions, PPI network was also constructed (Fig. 1C). Based on the median expression value, patients were assigned to high-expression and low-expression group. For investigating potential prognostic value of the 97 DEARGs, the corresponding survival data was assessed by Cox regression method. Figure 1D revealed LIHC patients with low NTF3, GNE, SEMA7A, GLUD1, PDK4 and RHOB expression were related to a worse prognosis, while LIHC patients with high expression of CCN6, EGF, TRAF2, SRC, UBE2C, CRYAB, ANXA2, ITGA2, MMP9, E2F1, NQO1, HK2, PTHLH, PBK, MAD2L1, CDKN2A, SFN, BUB1, CDC25C, HMGA1, ETV4, CDK1, BSG, PLK1, SPP1, BIRC5, and EZH2 showed a worse prognosis (all *P* value < .05). Thus, these 33 DEARGs were identified as prognostic-related DEARGs.

### 3.2. Functional enrichment analysis of DEARGs

**Figure 2.**
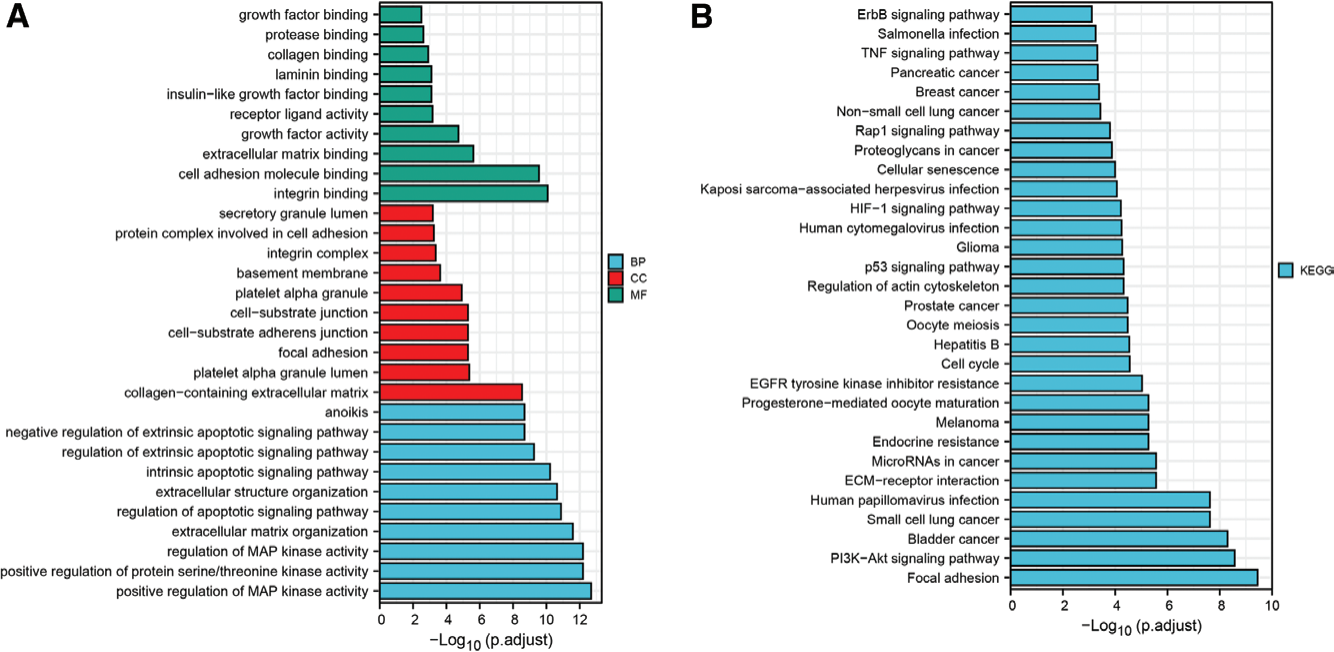
Functional enrichment analysis of the 97 DEARGs. (A) The bar plot of GO analysis, including BP, CC and MF analysis. (B) The bar plot of KEGG pathway analysis. DEARG = differentially expressed anoikis-related gene, GO = gene ontology, KEGG = Kyoto Encyclopedia of Genes and Genomes.

The above expression analysis indicated that 97 ARGs were differentially expressed in LIHC tissues. To predict their potential biological functions and molecular mechanisms, functional enrichment analyses were conducted. In Figure 2A, GO analysis, including BP, CC and MF analysis, illustrated these DEARGs were mainly associated with regulation of MAP kinase activity, regulation of protein serine/threonine kinase activity, regulation of apoptotic signaling pathway and anoikis. KEGG pathway analysis revealed these DEARGs were primarily correlated with the process of PI3K-Akt signaling pathway, ECM-receptor interaction, endocrine resistance, progesterone-mediated oocyte maturation and EGFR tyrosine kinase inhibitor resistance (Fig. 2B). The results of functional enrichment analysis further indicated their correlations with anoikis.

### 3.3. The expression levels and genetic mutation overview of prognostic-related DEARGs in LIHC

**Figure 3.**
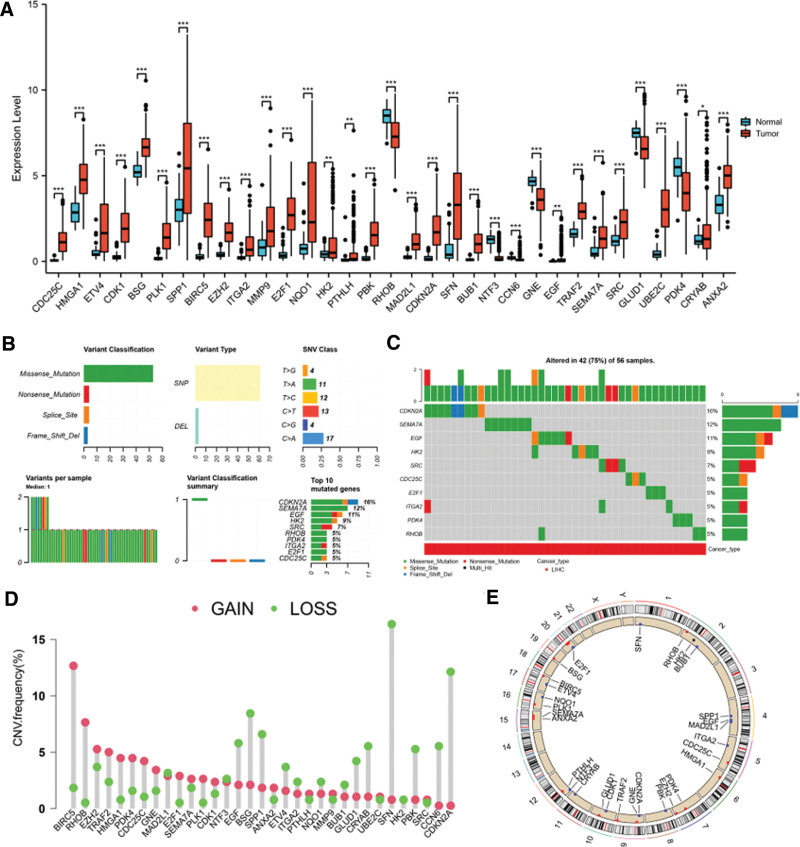
Expression status and mutation overviews of the 33 prognostic-related ARGs in LIHC. (A) The mRNA levels of prognostic-related ARGs in LIHC and normal liver tissues. (B-C) The mutation frequency and classification of prognostic-related ARGs in LIHC. (D) The CNV variation frequency of prognostic-related ARGs in LIHC. The height of the column represented the alteration frequency. (E) The location of CNV alteration of prognostic-related ARGs on chromosomes in LIHC. Note: *P < .05, **P < .01, ***P < .001. ARG = anoikis-related gene. CNV = copy number variation, LIHC = liver hepatocellular carcinoma.

The expression landscape of the 33 prognostic-related DEARGs in normal and LIHC tissues were visualized in Figure 3A. More specifically, the expression levels of CDC25C, HMGA1, ETV4, CDK1, BSG, PLK1, SPP1, BIRC5, EZH2, ITGA2, MMP9, E2F1, NQO1, HK2, PTHLH, PBK, MAD2L1, CDKN2A, SFN, BUB1, EGF, TRAF2, SEMA7A, SRC, UBE2C, CRYAB and ANXA2 were increased in LIHC tissues, while the expression levels of RHOB, NTF3, CCN6, GNE, GLUD1 and PDK4 were decreased in LIHC tissues (all *P* value < .05). The CNV and SNV status of 33 prognostic-related DEARGs in LIHC were also analyzed. Genetic mutations were detected in 42 (75%) of 56 LIHC samples. Figure 3B and 3C showed that gene mutation classifications included missense mutation, frame-shift deletion, splice-site variation and nonsense mutation. Missense mutation was the most common gene mutation classification. As for variant type and SNV class, SNP was the most common type and C>A ranked the top, respectively. Moreover, CDKN2A was correlated with highest mutation frequency in the 33 prognostic-related DEARGs. We also summarized the CNV frequency of these 33 genes (Fig. 3D). CNV losses were detected in CCN6, CDKN2A, SFN, PBK, CRYAB, BUB1, GLUD1, NQO1, ETV4, ITGA2, SPP1, BSG, EGF, NTF3 and MAD2L1, while CNV gains were detected in RHOB, BIRC5, EZH2, TRAF2, HMGA1, PDK4, CDC25C, GNE, E2F1, SEMA7A, CDK1, PLK1, ANXA2, PTHLH, MMP9, UBE2C, and SRC. But no significance CNV alteration was detected in HK2. In addition, we also visualized the positions of the CNV on chromosomes in Figure 3E.

### 3.4. Establishment and validation of an anoikis-related prognostic signature

**Figure 4.**
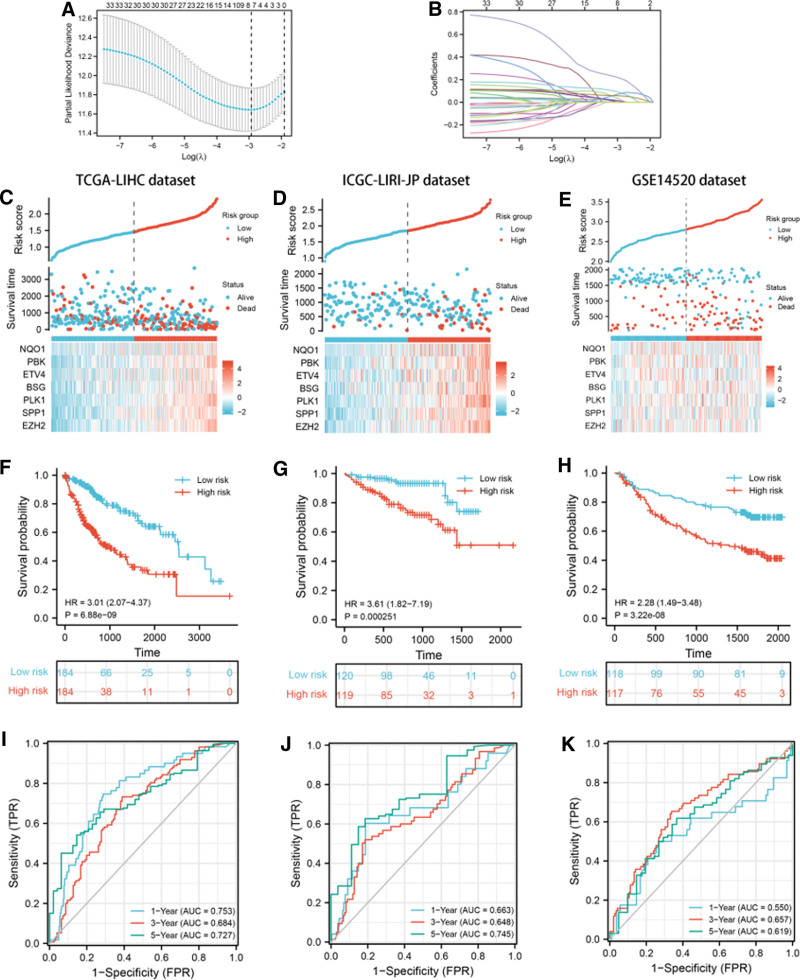
Prognostic model constructed by the 33 prognostic-related ARGs. The partial likelihood deviance (A) and coefficients (B) of the prognostic model. Risk score distribution, patients’ survival status and expression status of the 7 ARGs associated with risk score in TCGA-LIHC dataset (C), in ICGC-LIRI-JP dataset (D) and GSE14520 dataset (E). The OS plots and corresponding ROC curves of high-risk group and low-risk group in TCGA-LIHC dataset (F, I), in ICGC-LIRI-JP dataset (G, J) and GSE14520 dataset (H, K). ARG = anoikis-related gene, ICGC = International Cancer Genome Consortium, LIHC = liver hepatocellular carcinoma, TCGA = The Cancer Genome Atlas.

**Figure 5.**
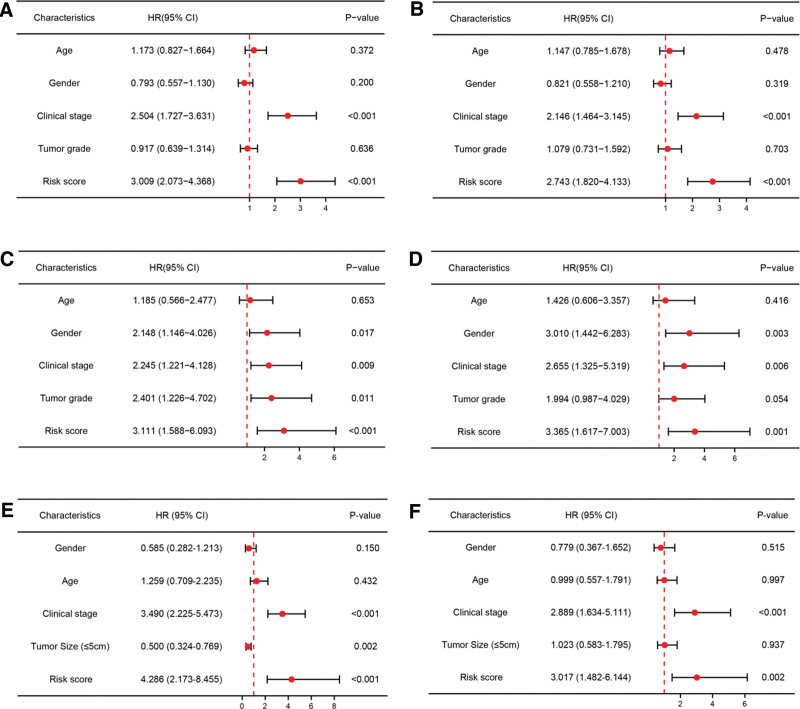
Univariate and multivariate Cox regression analysis considering clinical characteristics and risk score in TCGA-LIHC dataset (A-B), in ICGC-LIRI-JP dataset (C-D) and GSE14520 dataset (E-F). ICGC = International Cancer Genome Consortium, LIHC = liver hepatocellular carcinoma, TCGA = The Cancer Genome Atlas.

**Figure 6.**
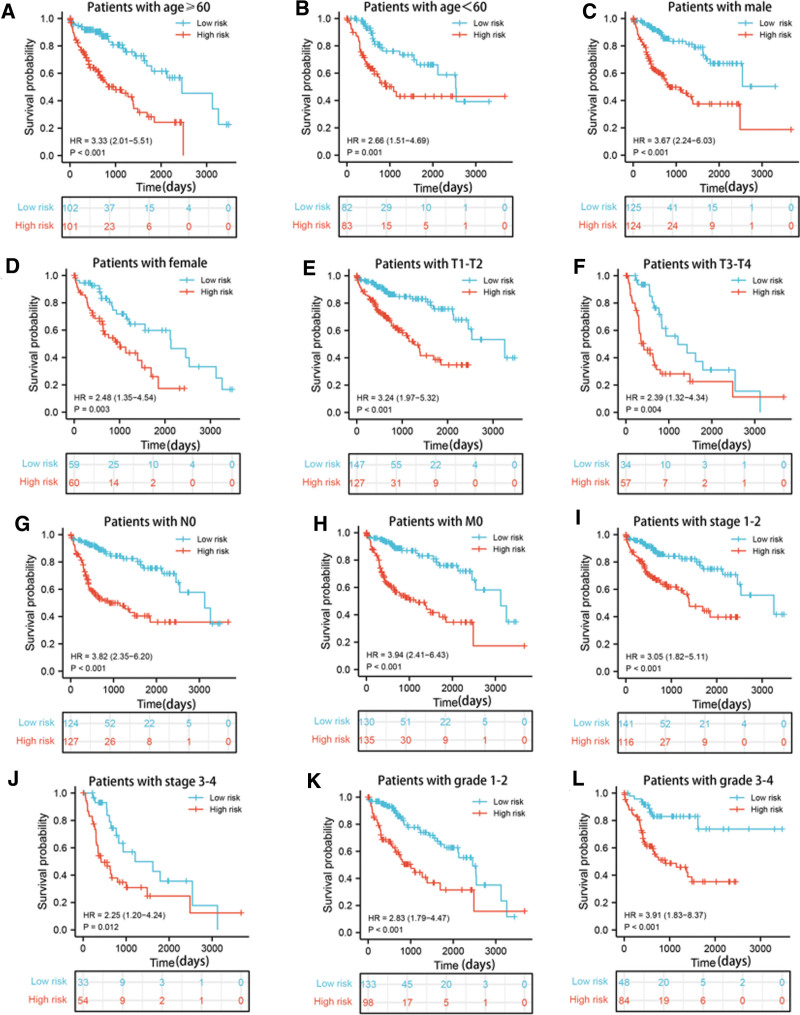
The OS curves of high-risk group and low-risk group based on the subgroups of age (A, B), gender (C, D), T stage (E, F), N stage (G) and M stage (H) clinical stage (I, J) and tumor grade (K, L).

For better predicting LIHC patients’ prognosis, LASSO Cox regression method was utilized to analyze the 33 prognostic-related DEARGs in the abovementioned survival analyses, and finally identified 7 genes (BSG, ETV4, EZH2, PLK1, PBK, NQO1, and SPP1) for prognostic risk model construction. In Figure 4A and 4B, partial likelihood deviance and coefficients of the prognostic risk model were shown. Risk score = (0.086379)*BSG expression + (0.011277)*ETV4 expression + (0.214123)*EZH2 expression + (0.074863)*PLK1 expression + (0.026396)*PBK expression + (0.010835)*NQO1 expression + (0.057033)*SPP1 expression. Patients were divided into high-risk and low-risk group based on median risk score. The risk score distribution, survival status and expression status of each sample in TCGA-LIHC dataset, ICGC-LIRI-JP dataset and GSE14520 were illustrated in Figure 4C, 4D, and 4E, respectively. In TCGA-LIHC dataset, high-risk group showed a worse prognosis compared to low-risk group (*P* value = 6.88e-09, Fig. 4F). The 1-year, 3-year and 5-year AUC values were 0.753, 0.684 and 0.727, respectively (Fig. 4I). Next, ICGC-LIRI-JP dataset and GSE14520 dataset were utilized to validate predictive performance of the prognostic signature. Similar survival outcomes also existed in ICGC-LIRI-JP dataset (*P* value = .000251, Fig. 4G) and GSE14520 dataset (*P* value = 3.22e-08, Fig. 4H). The 1-year, 3-year and 5-year AUC values of ICGC-LIRI-JP dataset were 0.663, 0.648 and 0.745 (Fig. 4J), while the 1-year, 3-year and 5-year AUC values of GSE14520 dataset were 0.550, 0.657, and 0.619 (Fig. 4K). To explore the potential factors that affected LIHC patients’ prognosis, univariate and multivariate analysis were conducted in TCGA-LIHC dataset (Fig. 5A and 5B), ICGC-LIRI-JP dataset (Fig. 5C and 5D) and GSE14520 dataset (Fig. 5E and 5F), which identified risk score and clinical stage as independent risk factors. Moreover, we also explored predictive performance of the prognostic model in patients with distinct clinical characteristics. In patients with age ≥ 60 years (Fig. 6A, *P* value<.001), Patients with age<60 years (Fig. 6B, *P* value = .001), male patients (Fig. 6C, *P* value<.001), female patients (Fig. 6D, *P* value = .003), patients with T1-2 (Fig. 6E, *P* value<.001), patients with T3–4 (Fig. 6F, *P* value = .004), patients with N0 (Fig. 6G, *P* value<.001), patients with M0 (Fig. 6H, *P* value<.001), patients with clinical stage 1–2 (Fig. 6I, *P* value<.001), patients with clinical stage 3–4 (Fig. 6J, *P* value = .012), patients with tumor grade 3–4 (Fig. 6K, *P* value<.001) and patients with tumor grade 3–4 (Fig. 6L, *P* value<.001), LIHC patients in high-risk group were related to unfavorable prognosis.

### 3.5. Risk score correlated with clinical characteristics

**Figure 7.**
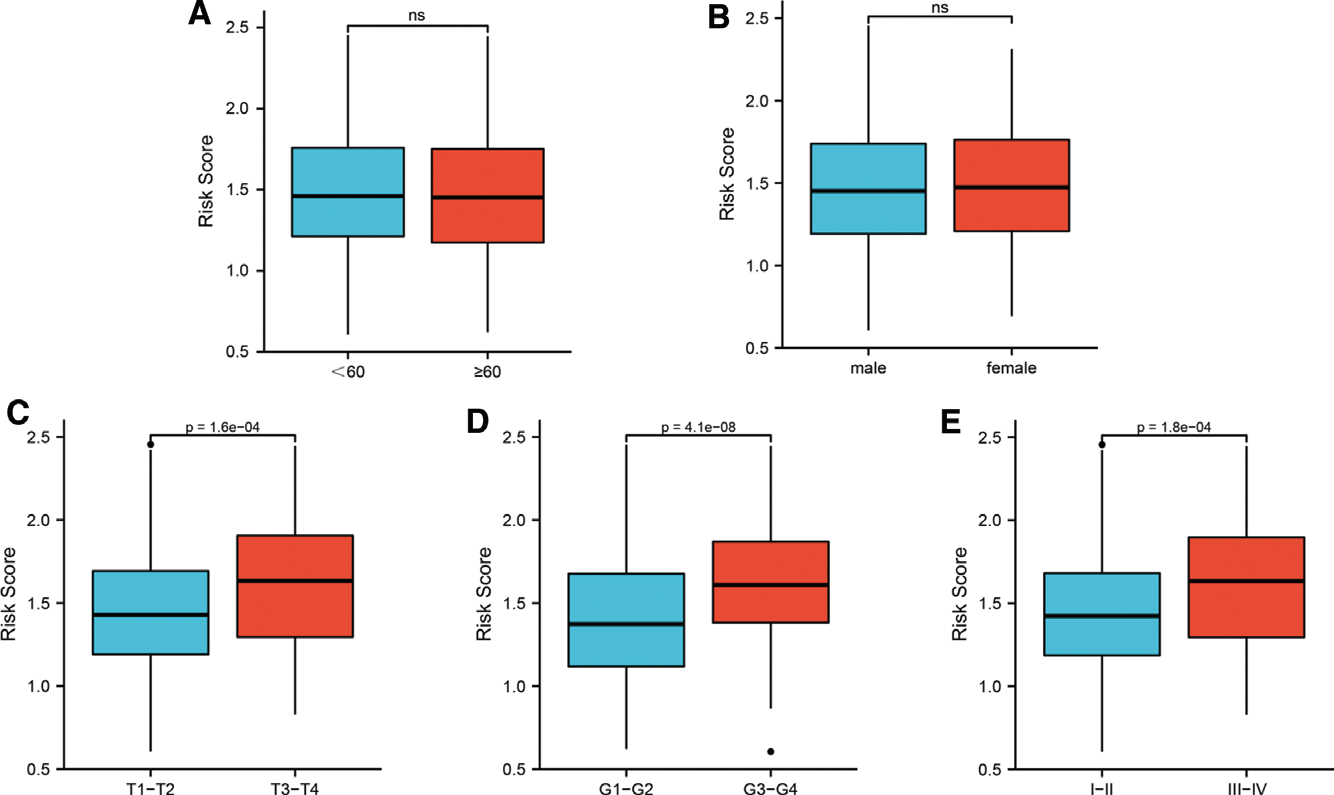
Risk score related to clinical features in LIHC. The difference of risk score in subgroups of age (A), gender (B), T stage (C), tumor grade (D) and clinical stage (E). LIHC = liver hepatocellular carcinoma.

The influence of clinical characteristics on risk score was also analyzed. According to patients’ gender, age, T stage, tumor grade and clinical stage, patients were assigned to distinct subgroups to compare their risk score. In the subgroup of age (≥60 years or not) and gender, no statistical significance was detected between the 2 group (Fig. 7A and 7B, all *P* value>.05). However, patients with T3-T4 stage had higher risk score than those with T1-T2 stage (Fig. 7C, *P* value = 1.6e-04), and LIHC patients with tumor grade 3–4 showed higher risk score than those with tumor grade 1–2 (Fig. 7D, *P* value = 4.1e-08). In addition, compared with patients with clinical stage 1–2, patients with clinical stage 3–4 also revealed higher risk score (Fig. 7E, *P* value = 1.8e-04).

### 3.6. Risk score correlated with immune cell infiltration

**Figure 8.**
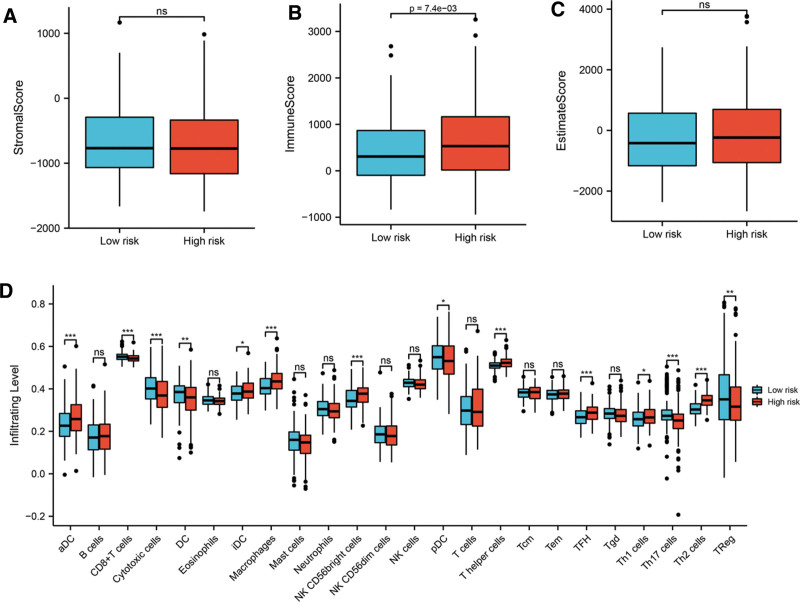
The association between risk score and immune cell infiltration. No statistical difference was found in StromalScore (A) and ESTIMATEScore (C) between high-risk and low-risk group. While high-risk group had a higher ImmuneScore (B) than low-risk group. (D) The infiltrating levels of 24 kinds of immunecytos in the 2 groups. Note: *P < .05, **P < .01, ***P < .001.

Tumor immune microenvironment was considered as one of the factors that influenced tumor biological behavior. For exploring the relationship between risk score and immune cell infiltration, we first utilized the ESTIMATE algorithm to calculate StromalScore, ImmuneScore and EstimateScore in high-risk and low-risk group. The result revealed higher ImmuneScore was detected in high-risk group (Fig. 8B, *P* value = 7.4e-03). As for StromalScore (Fig. 8A, *P* value>.05) and EstimateScore (Fig. 8C, *P* value>.05), no statistical significance was observed between high-risk and low-risk group. Afterward, according to ssGSEA algorithm, the infiltrating levels of 24 kinds of immunocytes in the 2 groups were compared. As shown in Figure 8D, the infiltrating levels of activated dendritic cell, immature dendritic cell, macrophages, NK CD56bright cells, T helper cells, TFH cells, Th1 cells and Th2 cells were upregulated in high-risk group, while the infiltrating levels of CD8 + T cells, cytotoxic cells, dendritic cell (DC), plasmacytoid dendritic cell (pDC), Th17 cells and Treg cells were decreased in high-risk group. No significant difference was detected in infiltrating levels of B cells, eosinophils, mast cells, neutrophils, NK cells, NK CD56dim cells, T cells, Tcm cells, Tem cells and Tgd cells between the 2 groups.

### 3.7. Risk score correlated with the expression of immune checkpoint

**Figure 9.**
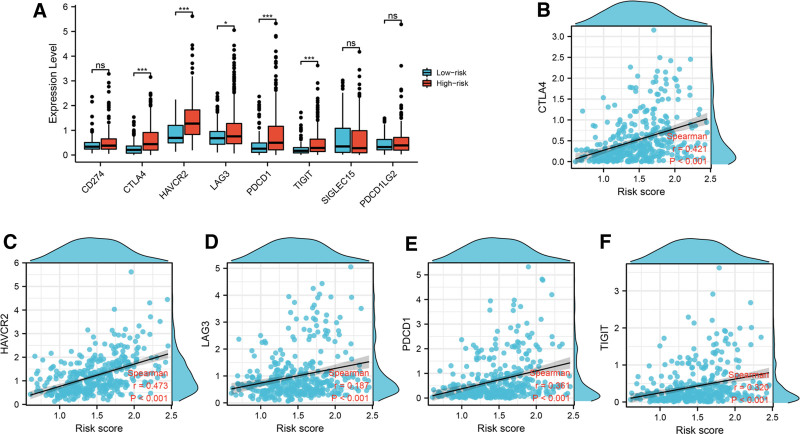
Risk score correlated with immune checkpoints expression. (A) The expression levels of common immune checkpoints in high-risk and low-risk groups. The correlation between CTLA4 (B), HAVCR2 (C), LAG3 (D), PDCD1 (E) and TIGIT (F) expression and risk score in LIHC. Note: *P < .05, ***P < .001. LIHC = liver hepatocellular carcinoma.

Currently, checkpoint inhibitor-based immunotherapy played a crucial role in cancer immunotherapy.^[20]^ Thus, we investigated immune checkpoint response status in the 2 groups. As illustrated in Figure 9A, the expression levels of CTLA4, HAVCR2, LAG3, PDCD1 and TIGIT were significantly upregulated in high-risk group. Nevertheless, there was no significant difference in CD274, SIGLEC15 and PDCD1LG2 expression between the 2 group. Next, using spearman correlation analysis, the current study visualized the association between risk score and CTLA4 (cor = 0.421, *P* value<.001), HAVCR2 (cor = 0.473, *P* value<.001), LAG3 (cor = 0.187, *P* value<.001), PDCD1 (cor = 0.361, *P* value<.001) and TIGIT (cor = 0.320, *P* value<.001) expression, which indicated the expression levels of these 5 immune checkpoints were positively correlated with risk score (Fig. 9B–9F). These results revealed that high-risk group was likely to generate immunosuppressive microenvironment.

### 3.8. Validation of the expression status of risk score-associated ARGs in LIHC

**Figure 10.**
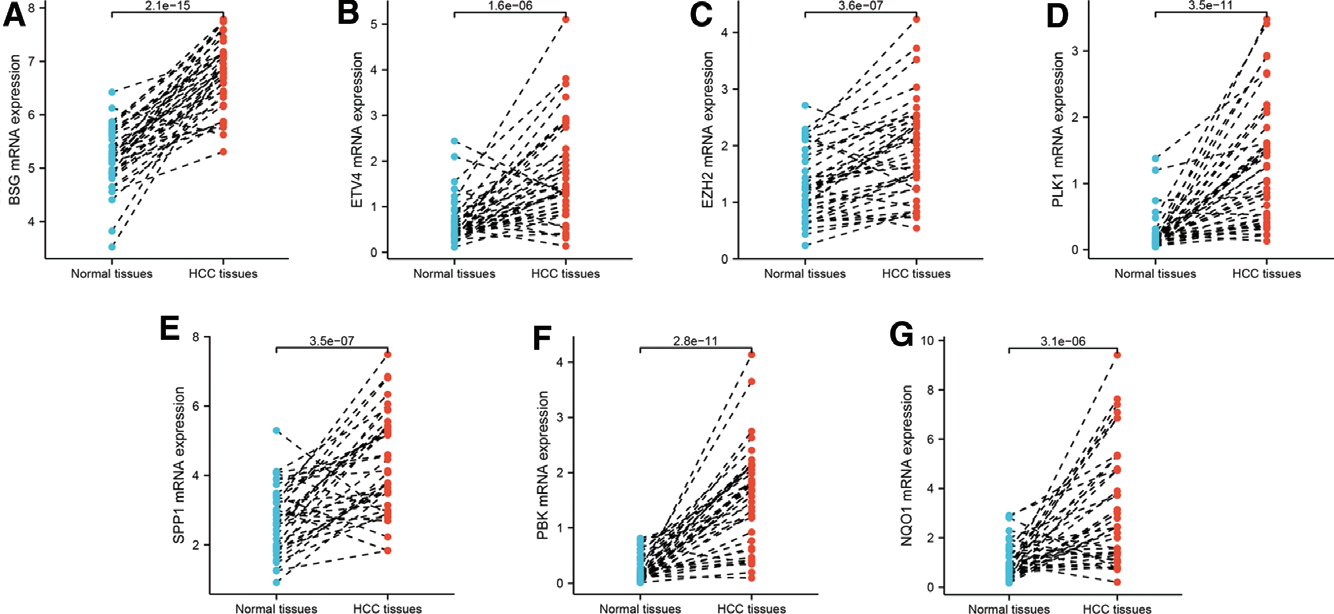
Validation of the transcription levels of risk score-related ARGs by qRT-PCR. Compared with paired normal liver tissues, the mRNA levels of BSG (A), ETV4 (B), EZH2 (C), PLK1 (D), SPP1 (E), PBK (F) and NQO1 (G) in LIHC tissues were significantly higher. ARG = anoikis-related gene, LIHC = liver hepatocellular carcinoma, qRT-PCR = quantitative real-time polymerase chain reaction.

**Figure 11.**
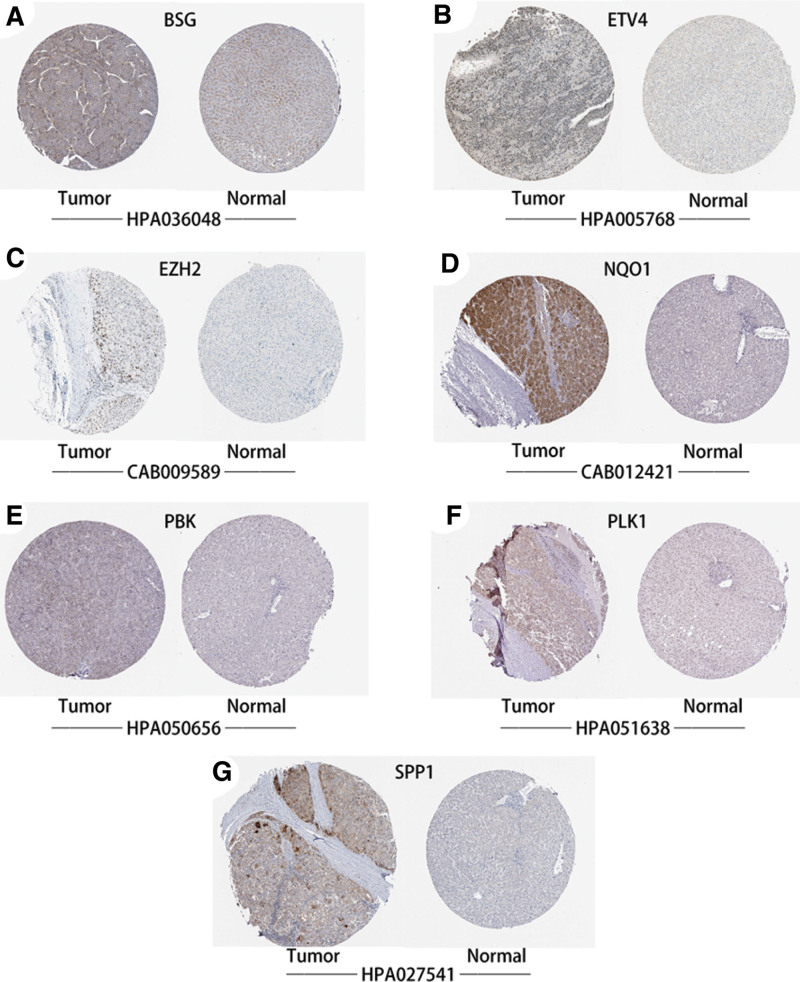
Validation of the protein levels of risk score-related ARGs by IHC staining. BSG (A), ETV4 (B), EZH2 (C), NQO1 (D), PBK (E), PLK1 (F) and SPP1 (G) in LIHC tissues and normal liver tissues. ARG = anoikis-related gene, LIHC = liver hepatocellular carcinoma.

Using qRT-PCR, the present study validated the mRNA level of risk score-associated ARGs in LIHC tissues and normal liver tissues. As expected, the mRNA levels of BSG (Fig. 10A, *P* value = 2.1e-15), ETV4 (Fig. 10B, *P* value = 1.6e-06), EZH2 (Fig. 10C, *P* value = 3.6e-07), PLK1 (Fig. 10D, *P* value = 3.5e-11), SPP1 (Fig. 10E, *P* value = 3.5e-07), PBK (Fig. 10F, *P* value = 2.8e-11) and NQO1 (Fig. 10G, *P* value = 3.1e-06) were significantly increased in LIHC tissues compared to those in normal liver tissues. As for protein level, immunohistochemical (IHC) staining also verified the expression levels of BSG (Fig. 11A), ETV4 (Fig. 11B), EZH2 (Fig. 11C), NQO1 (Fig. 11D), PBK (Fig. 11E), PLK1 (Fig. 11F) and SPP1 (Fig. 11G) were significantly increased in LIHC tissues. The above results were consistent to those in TCGA-LIHC dataset.

## 4. Discussion

LIHC is the malignant tumor with high rates of recurrence and metastasis, which causes a disproportionate health burden to patients.^[21]^ The dismal overall 5-year survival rate of <20% is partly due to metastasis of LIHC.^[3]^ As a specific form of cell apoptosis, previous studies suggested anoikis resistance of detached cancer cells is a key process of metastasis and is a hallmark of metastasis.^[22]^ Some of the ARGs were also identified as prognostic indicators and regulated occurrence and progression of corresponding cancer cells.^[11–14]^ With the development of sequencing technique and establishment of genome databases, large-scale and comprehensive analyses on ARGs in LIHC become feasible. However, comprehensive bioinformatic analyses focusing on the expression status and prognostic performance of ARGs in LIHC as well as their associations with tumor immune microenvironment is lacking.

We firstly explored the mRNA levels of ARGs in LIHC tissues and normal liver tissues. In the expression analysis, 97 genes (67 upregulated genes and 30 downregulated genes) were differentially expressed. These 97 DEARGs were next enrolled in functional enrichment analysis. The result revealed these genes were mainly involved in regulation of MAP kinase activity, regulation of protein serine/threonine kinase activity, regulation of apoptotic signaling pathway, anoikis, PI3K-Akt signaling pathway, ECM-receptor interaction and EGFR tyrosine kinase inhibitor resistance. In previous publications, activation of PI3K/Akt signaling pathway was considered as the most common form to result in anoikis resistance in cancer cells. On the one hand, through regulating phosphorylation of pro-apoptotic proteins, such as BAD and pro-CASP9, activation of Akt could directly inhibit their functions.^[23,24]^ On the other hand, sustained Akt activation was often accompanied by elevated N-cadherin expression. N-cadherin induced anoikis resistance by recruiting PI3K.^[25]^ Cancer cells could adapt to the loss of anchorage and generate anoikis resistance by activating EGFR family members^[26]^ or MAPK/ERK signaling pathway-mediated suppression of BIM.^[26,27]^ In addition, anoikis was an apoptotic process indeed, so it was also modulated by both intrinstic and extrinsic apoptotic pathways.^[28]^ These abovementioned perspectives provided powerful support for the results of GO and KEGG pathway analysis.

The 97 DEARGs were enrolled to perform survival analysis, and 33 prognostic-related DEARGs were selected out. Risk score in the prognostic signature consisted of BSG, ETV4, EZH2, NQO1, PLK1, PBK and SPP1. Patients with high risk score in TCGA-LIHC cohort were associated with unfavorable prognosis. ICGC-LIRI-JP cohort and GSE14520 cohort were downloaded for external validating the prognostic model, and showed similar results with TCGA-LIHC cohort. According to the results of Time ROC analysis, the prediction model showed moderate to high predictive accuracy on the whole. Another bioinformatic analysis building up ARGs-related prognostic signature for patients with endometrial carcinoma also indicated an unfavorable prognosis in high-risk group.^[29]^ Nevertheless, large-scale and multi-center studies are required to verify these results.

TME in LIHC is generally thought to be immunosuppressive, which allows cancer cells to evade anti-cancer immunity and promote tumor growth and metastasis.^[30]^ For instance, cancer cells evaded immune surveillance by inhibiting functions of DC.^[31]^ FOXP3 + Tregs inhibited the production and activation of anti-tumor effector cells, thus generating immunosuppressive functions in TME.^[32]^ In our study, the results indicated that the upregulated infiltrating levels of activated dendritic cell, immature dendritic cell, macrophages, NK CD56bright cells, T helper cells, TFH cells, Th1 cells and Th2 cells in high-risk group, while the decreased infiltrating levels of CD8 + T cells, cytotoxic cells, DC, pDC, Th17 cells and Treg cells in high-risk group. High-risk group also showed higher ImmuneScore. The result indicated there were differences in TME between the 2 groups. Currently, immune checkpoint inhibitor (ICI) therapy is considered as a solution to treat unresectable cancer and is one of adjuvant therapies after surgery.^[33]^ The current study showed CTLA4, HAVCR2, LAG3, PDCD1, and TIGIT expression were significantly elevated in high-risk group and were positively correlated with risk score. According to the theory of “hot” tumor and “cold” tumor,^[34]^ high-risk group might be the “hot” tumor and tend to profit from ICI treatment.

Using qRT-PCR and IHC staining data from HPA website, higher expression levels of the 7 risk score-related ARGs in LIHC tissues, including BSG, ETV4, EZH2, NQO1, PLK1, PBK and SPP1, were detected. In previous publications, EZH2, a cancer-associated protein with histone-methyltransferase activity, was overexpressed and was related to poor prognosis in LIHC.^[35]^ As for NQO1,^[36]^ PLK1^[37]^ and BSG,^[38]^ the prognostic performance and expression status were similar with EZH2. Wang et al suggested SPP1 was upregulated in LIHC tissues and multiple LIHC cell lines and enhanced LIHC cell growth through targeting miR-181c.^[39]^ Yang et al reported overexpression of PBK promotes metastasis of LIHC by regulating ETV4-uPAR signaling pathway and PBK expression was increased in LIHC tissues both on mRNA and protein level.^[40]^ In addition, the overexpression of ETV4 was also detected in LIHC cell lines and tissues, and in vivo and in vitro experiments revealed ETV4 promoted migration and invasion of LIHC cells.^[41]^ To some extent, the above studies showed similar expression status of the 7 ARGs in LIHC with the current study.

Some limitations also should be acknowledged. Firstly, most of the analyses were performed on mRNA level, some results might be inapplicable to studies on protein level. Secondly, the underlying mechanisms of ARGs-related signature to influence prognosis, immune cell infiltration and immune checkpoints should be further studied by experiments. Thirdly, many factors, such as race, age and gender, also influenced patients’ genetic background, more in-depth studies were required to study this field.

## 5. Conclusions

In all, our study established an anoikis-related gene signature for LIHC patients. It was conducive to prognosis prediction of LIHC patients and reflected immune cell infiltration and immune checkpoints response in patients with LIHC. All these results offered novel insights on prognostic evaluation and individualized immunotherapy for LIHC patients.

## Author contributions

**Conceptualization:** Dianqian Wang.

**Funding acquisition:** Yunsheng Qin.

**Investigation:** Dongxiao Ding.

**Methodology:** Dianqian Wang.

**Project administration:** Dongxiao Ding, Yunsheng Qin.

**Visualization:** Dianqian Wang.

**Writing – original draft:** Dongxiao Ding.

**Writing – review & editing:** Dianqian Wang, Yunsheng Qin.

## Supplementary Material




