# Supplementary material for: Development and validation of multi-omic prognostic signature of anoikis-related genes in liver hepatocellular carcinoma
Source: Medicine (Baltimore). 2023 Nov 17;102(46):e36190. doi: 10.1097/MD.0000000000036190 (PMC10659623; doi:10.1097/MD.0000000000036190)
Supplement: Supplementary file 1 [file medi-102-e36190-s001.docx]

Supplementary table 1. The list of 404 anoikis-related genes.

| Gene Symbol | Description | Relevance score |
| --- | --- | --- |
| BRMS1 | BRMS1 Transcriptional Repressor And Anoikis Regulator | 14.61328793 |
| PTK2 | Protein Tyrosine Kinase 2 | 7.272868156 |
| NTRK2 | Neurotrophic Receptor Tyrosine Kinase 2 | 7.251225471 |
| BCL2L11 | BCL2 Like 11 | 6.679876804 |
| SRC | SRC Proto-Oncogene, Non-Receptor Tyrosine Kinase | 6.172210693 |
| CEACAM6 | CEA Cell Adhesion Molecule 6 | 6.095650196 |
| CAV1 | Caveolin 1 | 5.452927589 |
| AKT1 | AKT Serine/Threonine Kinase 1 | 5.397092819 |
| ITGB1 | Integrin Subunit Beta 1 | 4.993362904 |
| CEACAM5 | CEA Cell Adhesion Molecule 5 | 4.650313377 |
| EGFR | Epidermal Growth Factor Receptor | 4.605351448 |
| BCL2 | BCL2 Apoptosis Regulator | 4.532852173 |
| CASP8 | Caspase 8 | 4.478847504 |
| PTRH2 | Peptidyl-TRNA Hydrolase 2 | 4.185810566 |
| STAT3 | Signal Transducer And Activator Of Transcription 3 | 4.131558418 |
| SIK1 | Salt Inducible Kinase 1 | 4.065357208 |
| TLE1 | TLE Family Member 1, Transcriptional Corepressor | 4.063039303 |
| DAPK2 | Death Associated Protein Kinase 2 | 3.984466791 |
| CTNNB1 | Catenin Beta 1 | 3.971251249 |
| ZNF304 | Zinc Finger Protein 304 | 3.936246872 |
| MAPK1 | Mitogen-Activated Protein Kinase 1 | 3.726213217 |
| BMF | Bcl2 Modifying Factor | 3.726126432 |
| ITGA5 | Integrin Subunit Alpha 5 | 3.671030045 |
| TP53 | Tumor Protein P53 | 3.631812096 |
| MCL1 | MCL1 Apoptosis Regulator, BCL2 Family Member | 3.572852612 |
| BCL2L1 | BCL2 Like 1 | 3.356115103 |
| CASP3 | Caspase 3 | 3.11302495 |
| CDH1 | Cadherin 1 | 3.05269289 |
| BAD | BCL2 Associated Agonist Of Cell Death | 2.956306934 |
| PIK3CA | Phosphatidylinositol-4,5-Bisphosphate 3-Kinase Catalytic Subunit Alpha | 2.937769175 |
| PAK1 | P21 (RAC1) Activated Kinase 1 | 2.924462318 |
| ITGAV | Integrin Subunit Alpha V | 2.868912697 |
| FN1 | Fibronectin 1 | 2.815199375 |
| MAPK3 | Mitogen-Activated Protein Kinase 3 | 2.723765612 |
| PTGS2 | Prostaglandin-Endoperoxide Synthase 2 | 2.686130762 |
| BAX | BCL2 Associated X, Apoptosis Regulator | 2.542374134 |
| BCAR1 | BCAR1 Scaffold Protein, Cas Family Member | 2.542374134 |
| PTEN | Phosphatase And Tensin Homolog | 2.513079405 |
| ERBB2 | Erb-B2 Receptor Tyrosine Kinase 2 | 2.428184032 |
| PDK4 | Pyruvate Dehydrogenase Kinase 4 | 2.41479063 |
| ANGPTL4 | Angiopoietin Like 4 | 2.393246174 |
| CYCS | Cytochrome C, Somatic | 2.33425355 |
| BRAF | B-Raf Proto-Oncogene, Serine/Threonine Kinase | 2.330135345 |
| YAP1 | Yes1 Associated Transcriptional Regulator | 2.326847553 |
| ANKRD13C | Ankyrin Repeat Domain 13C | 2.32548666 |
| ITGA2 | Integrin Subunit Alpha 2 | 2.292931557 |
| ANXA5 | Annexin A5 | 2.260920763 |
| BIRC5 | Baculoviral IAP Repeat Containing 5 | 2.250733852 |
| MTOR | Mechanistic Target Of Rapamycin Kinase | 2.246199608 |
| TIMP1 | TIMP Metallopeptidase Inhibitor 1 | 2.24024272 |
| BDNF | Brain Derived Neurotrophic Factor | 2.216442585 |
| CSPG4 | Chondroitin Sulfate Proteoglycan 4 | 2.190145969 |
| BSG | Basigin (Ok Blood Group) | 2.190145969 |
| AKT2 | AKT Serine/Threonine Kinase 2 | 2.177864075 |
| STK11 | Serine/Threonine Kinase 11 | 2.147129774 |
| IGF1 | Insulin Like Growth Factor 1 | 2.142858028 |
| IGF1R | Insulin Like Growth Factor 1 Receptor | 2.141947269 |
| ITGA6 | Integrin Subunit Alpha 6 | 2.105042458 |
| ILK | Integrin Linked Kinase | 2.078502417 |
| CFLAR | CASP8 And FADD Like Apoptosis Regulator | 2.078392744 |
| RHOA | Ras Homolog Family Member A | 2.063979149 |
| HIF1A | Hypoxia Inducible Factor 1 Subunit Alpha | 2.060393333 |
| DAP3 | Death Associated Protein 3 | 2.050726175 |
| MYBBP1A | MYB Binding Protein 1a | 2.018019438 |
| TLE5 | TLE Family Member 5, Transcriptional Modulator | 1.995359778 |
| ITGA3 | Integrin Subunit Alpha 3 | 1.993474841 |
| PTK2B | Protein Tyrosine Kinase 2 Beta | 1.990646482 |
| CCND1 | Cyclin D1 | 1.977019191 |
| CTTN | Cortactin | 1.977019191 |
| CALR | Calreticulin | 1.940452576 |
| ATF4 | Activating Transcription Factor 4 | 1.940452576 |
| CDCP1 | CUB Domain Containing Protein 1 | 1.927523375 |
| CHEK2 | Checkpoint Kinase 2 | 1.904656887 |
| SKP2 | S-Phase Kinase Associated Protein 2 | 1.903686523 |
| HGF | Hepatocyte Growth Factor | 1.879078865 |
| E2F1 | E2F Transcription Factor 1 | 1.877498746 |
| EGF | Epidermal Growth Factor | 1.866675496 |
| PIK3CG | Phosphatidylinositol-4,5-Bisphosphate 3-Kinase Catalytic Subunit Gamma | 1.863395691 |
| ITGB4 | Integrin Subunit Beta 4 | 1.851416349 |
| DAPK1 | Death Associated Protein Kinase 1 | 1.841562867 |
| PIK3R1 | Phosphoinositide-3-Kinase Regulatory Subunit 1 | 1.81670022 |
| PIK3R3 | Phosphoinositide-3-Kinase Regulatory Subunit 3 | 1.813362122 |
| MAP2K1 | Mitogen-Activated Protein Kinase Kinase 1 | 1.792118907 |
| CXCL12 | C-X-C Motif Chemokine Ligand 12 | 1.772871852 |
| LGALS3 | Galectin 3 | 1.737552643 |
| FBXW7-AS1 | FBXW7 Antisense RNA 1 | 1.729995251 |
| BAK1 | BCL2 Antagonist/Killer 1 | 1.725968122 |
| ABHD4 | Abhydrolase Domain Containing 4, N-Acyl Phospholipase B | 1.702597022 |
| CD44 | CD44 Molecule (Indian Blood Group) | 1.699145317 |
| ITGA4 | Integrin Subunit Alpha 4 | 1.685885668 |
| FADD | Fas Associated Via Death Domain | 1.685885668 |
| PHLDA2 | Pleckstrin Homology Like Domain Family A Member 2 | 1.685885668 |
| TGFB1 | Transforming Growth Factor Beta 1 | 1.681775212 |
| HMCN1 | Hemicentin 1 | 1.681775212 |
| MMP2 | Matrix Metallopeptidase 2 | 1.668566108 |
| CEBPB | CCAAT Enhancer Binding Protein Beta | 1.668566108 |
| CEMIP | Cell Migration Inducing Hyaluronidase 1 | 1.668566108 |
| CDKN3 | Cyclin Dependent Kinase Inhibitor 3 | 1.663046837 |
| CBL | Cbl Proto-Oncogene | 1.650566459 |
| CASP9 | Caspase 9 | 1.650566459 |
| SFN | Stratifin | 1.650566459 |
| MTDH | Metadherin | 1.650566459 |
| PRKCA | Protein Kinase C Alpha | 1.631799936 |
| TNFRSF10B | TNF Receptor Superfamily Member 10b | 1.631799936 |
| CXCL8 | C-X-C Motif Chemokine Ligand 8 | 1.631799936 |
| MIR200C | MicroRNA 200c | 1.631799936 |
| AR | Androgen Receptor | 1.615061879 |
| CDKN2A | Cyclin Dependent Kinase Inhibitor 2A | 1.612159133 |
| MAPK8 | Mitogen-Activated Protein Kinase 8 | 1.612159133 |
| CPT1A | Carnitine Palmitoyltransferase 1A | 1.612159133 |
| PIK3CB | Phosphatidylinositol-4,5-Bisphosphate 3-Kinase Catalytic Subunit Beta | 1.612159133 |
| CLDN1 | Claudin 1 | 1.612159133 |
| MIR204 | MicroRNA 204 | 1.612159133 |
| MIR26A1 | MicroRNA 26a-1 | 1.612159133 |
| CDKN1A | Cyclin Dependent Kinase Inhibitor 1A | 1.591509104 |
| CDKN1B | Cyclin Dependent Kinase Inhibitor 1B | 1.591509104 |
| KLF12 | Kruppel Like Factor 12 | 1.591509104 |
| NTRK1 | Neurotrophic Receptor Tyrosine Kinase 1 | 1.569918633 |
| PLAU | Plasminogen Activator, Urokinase | 1.569676399 |
| MYC | MYC Proto-Oncogene, BHLH Transcription Factor | 1.569676399 |
| SMAD4 | SMAD Family Member 4 | 1.569676399 |
| MUC1 | Mucin 1, Cell Surface Associated | 1.569676399 |
| PLK1 | Polo Like Kinase 1 | 1.569676399 |
| PLAUR | Plasminogen Activator, Urokinase Receptor | 1.569676399 |
| LGALS1 | Galectin 1 | 1.569676399 |
| PYCARD | PYD And CARD Domain Containing | 1.569676399 |
| SESN2 | Sestrin 2 | 1.569676399 |
| ITGB3 | Integrin Subunit Beta 3 | 1.564779282 |
| KRAS | KRAS Proto-Oncogene, GTPase | 1.564779282 |
| THBS1 | Thrombospondin 1 | 1.546430707 |
| BID | BH3 Interacting Domain Death Agonist | 1.546430707 |
| HRAS | HRas Proto-Oncogene, GTPase | 1.531511188 |
| CDK11B | Cyclin Dependent Kinase 11B | 1.521456242 |
| CDK11A | Cyclin Dependent Kinase 11A | 1.521456242 |
| XIAP | X-Linked Inhibitor Of Apoptosis | 1.514585376 |
| PPARG | Peroxisome Proliferator Activated Receptor Gamma | 1.49429822 |
| IL6 | Interleukin 6 | 1.49429822 |
| MIR145 | MicroRNA 145 | 1.49429822 |
| CCR7 | C-C Motif Chemokine Receptor 7 | 1.464258313 |
| MSLN | Mesothelin | 1.464258313 |
| RAC1 | Rac Family Small GTPase 1 | 1.45817852 |
| GRHL2 | Grainyhead Like Transcription Factor 2 | 1.45817852 |
| NOTCH1 | Notch Receptor 1 | 1.436274052 |
| RHOG | Ras Homolog Family Member G | 1.433068037 |
| CCAR2 | Cell Cycle And Apoptosis Regulator 2 | 1.433068037 |
| NQO1 | NAD(P)H Quinone Dehydrogenase 1 | 1.430161476 |
| BIRC3 | Baculoviral IAP Repeat Containing 3 | 1.427599192 |
| MMP13 | Matrix Metallopeptidase 13 | 1.395306826 |
| FAS | Fas Cell Surface Death Receptor | 1.392450094 |
| MTA1 | Metastasis Associated 1 | 1.392450094 |
| MYO5A | Myosin VA | 1.389716506 |
| EDA2R | Ectodysplasin A2 Receptor | 1.389716506 |
| CCN6 | Cellular Communication Network Factor 6 | 1.389716506 |
| MMP9 | Matrix Metallopeptidase 9 | 1.371192217 |
| ABL1 | ABL Proto-Oncogene 1, Non-Receptor Tyrosine Kinase | 1.371192217 |
| MAPK11 | Mitogen-Activated Protein Kinase 11 | 1.371192217 |
| PTHLH | Parathyroid Hormone Like Hormone | 1.36747551 |
| PDGFB | Platelet Derived Growth Factor Subunit B | 1.350542307 |
| GLI2 | GLI Family Zinc Finger 2 | 1.350542307 |
| EZH2 | Enhancer Of Zeste 2 Polycomb Repressive Complex 2 Subunit | 1.34951365 |
| CXCR4 | C-X-C Motif Chemokine Receptor 4 | 1.340317488 |
| RIPK1 | Receptor Interacting Serine/Threonine Kinase 1 | 1.331556201 |
| HMGA1 | High Mobility Group AT-Hook 1 | 1.328709483 |
| SIK2 | Salt Inducible Kinase 2 | 1.328709483 |
| TNFSF10 | TNF Superfamily Member 10 | 1.328709483 |
| ANGPTL2 | Angiopoietin Like 2 | 1.310277581 |
| S100A4 | S100 Calcium Binding Protein A4 | 1.305463791 |
| ETV4 | ETS Variant Transcription Factor 4 | 1.305463791 |
| NTF3 | Neurotrophin 3 | 1.305463791 |
| MIR21 | MicroRNA 21 | 1.305463791 |
| MIR124-1 | MicroRNA 124-1 | 1.305463791 |
| HTRA1 | HtrA Serine Peptidase 1 | 1.280489445 |
| LATS1 | Large Tumor Suppressor Kinase 1 | 1.280489445 |
| CEACAM3 | CEA Cell Adhesion Molecule 3 | 1.280489445 |
| EIF2AK3 | Eukaryotic Translation Initiation Factor 2 Alpha Kinase 3 | 1.277470469 |
| LAMC2 | Laminin Subunit Gamma 2 | 1.277470469 |
| LAMA3 | Laminin Subunit Alpha 3 | 1.277470469 |
| LAMB3 | Laminin Subunit Beta 3 | 1.277470469 |
| CDH2 | Cadherin 2 | 1.257829666 |
| CSNK2A1 | Casein Kinase 2 Alpha 1 | 1.257829666 |
| EDIL3 | EGF Like Repeats And Discoidin Domains 3 | 1.257829666 |
| ZEB2 | Zinc Finger E-Box Binding Homeobox 2 | 1.253331304 |
| TLN1 | Talin 1 | 1.253331304 |
| EPHA2 | EPH Receptor A2 | 1.237179637 |
| SOD2 | Superoxide Dismutase 2 | 1.237179637 |
| SIRT3 | Sirtuin 3 | 1.237179637 |
| OLFM3 | Olfactomedin 3 | 1.237179637 |
| CLU | Clusterin | 1.223291397 |
| SPINK1 | Serine Peptidase Inhibitor Kazal Type 1 | 1.223291397 |
| CPEB2 | Cytoplasmic Polyadenylation Element Binding Protein 2 | 1.223291397 |
| NAT1 | N-Acetyltransferase 1 | 1.215346813 |
| TSG101 | Tumor Susceptibility 101 | 1.215346813 |
| MIR200A | MicroRNA 200a | 1.215346813 |
| MIR6744 | MicroRNA 6744 | 1.215346813 |
| SERPINA1 | Serpin Family A Member 1 | 1.209756613 |
| AKT3 | AKT Serine/Threonine Kinase 3 | 1.19210124 |
| RELA | RELA Proto-Oncogene, NF-KB Subunit | 1.19210124 |
| TNFRSF1A | TNF Receptor Superfamily Member 1A | 1.19210124 |
| AFP | Alpha Fetoprotein | 1.19210124 |
| FASLG | Fas Ligand | 1.19210124 |
| NOX4 | NADPH Oxidase 4 | 1.19210124 |
| ITGA8 | Integrin Subunit Alpha 8 | 1.19210124 |
| SATB1 | SATB Homeobox 1 | 1.19210124 |
| CD63 | CD63 Molecule | 1.19210124 |
| EEF1A1 | Eukaryotic Translation Elongation Factor 1 Alpha 1 | 1.19210124 |
| LTB4R2 | Leukotriene B4 Receptor 2 | 1.19210124 |
| PBK | PDZ Binding Kinase | 1.19210124 |
| MAVS | Mitochondrial Antiviral Signaling Protein | 1.19210124 |
| HRC | Histidine Rich Calcium Binding Protein | 1.19210124 |
| RHOB | Ras Homolog Family Member B | 1.189194679 |
| CCN2 | Cellular Communication Network Factor 2 | 1.189194679 |
| PPP1R13B | Protein Phosphatase 1 Regulatory Subunit 13B | 1.189194679 |
| PLG | Plasminogen | 1.183026791 |
| MET | MET Proto-Oncogene, Receptor Tyrosine Kinase | 1.181053758 |
| RAF1 | Raf-1 Proto-Oncogene, Serine/Threonine Kinase | 1.167126775 |
| PARP1 | Poly(ADP-Ribose) Polymerase 1 | 1.167126775 |
| PRKCQ | Protein Kinase C Theta | 1.167126775 |
| BRCA2 | BRCA2 DNA Repair Associated | 1.167126775 |
| RB1 | RB Transcriptional Corepressor 1 | 1.167126775 |
| DOCK1 | Dedicator Of Cytokinesis 1 | 1.167126775 |
| HAVCR2 | Hepatitis A Virus Cellular Receptor 2 | 1.167126775 |
| SP1 | Sp1 Transcription Factor | 1.167126775 |
| VTN | Vitronectin | 1.167126775 |
| INHBB | Inhibin Subunit Beta B | 1.167126775 |
| PDCD4 | Programmed Cell Death 4 | 1.167126775 |
| RANBP9 | RAN Binding Protein 9 | 1.167126775 |
| PRPF4B | Pre-MRNA Processing Factor 4B | 1.167126775 |
| SESN1 | Sestrin 1 | 1.167126775 |
| SESN3 | Sestrin 3 | 1.167126775 |
| CD24 | CD24 Molecule | 1.167126775 |
| ZBTB7A | Zinc Finger And BTB Domain Containing 7A | 1.167126775 |
| MIR141 | MicroRNA 141 | 1.167126775 |
| ELANE | Elastase, Neutrophil Expressed | 1.14874959 |
| KDR | Kinase Insert Domain Receptor | 1.139968634 |
| MDM2 | MDM2 Proto-Oncogene | 1.139968634 |
| NFE2L2 | NFE2 Like BZIP Transcription Factor 2 | 1.139968634 |
| PRKCI | Protein Kinase C Iota | 1.139968634 |
| ZEB1 | Zinc Finger E-Box Binding Homeobox 1 | 1.139968634 |
| HK2 | Hexokinase 2 | 1.139968634 |
| KL | Klotho | 1.139968634 |
| CRYAB | Crystallin Alpha B | 1.139968634 |
| EPHB6 | EPH Receptor B6 | 1.139968634 |
| FGF2 | Fibroblast Growth Factor 2 | 1.139968634 |
| LTF | Lactotransferrin | 1.139968634 |
| IQGAP1 | IQ Motif Containing GTPase Activating Protein 1 | 1.139968634 |
| MGAT5 | Alpha-1,6-Mannosylglycoprotein 6-Beta-N-Acetylglucosaminyltransferase | 1.139968634 |
| SDCBP | Syndecan Binding Protein | 1.139968634 |
| ABHD2 | Abhydrolase Domain Containing 2, Acylglycerol Lipase | 1.139968634 |
| SPIB | Spi-B Transcription Factor | 1.139968634 |
| TRIM31 | Tripartite Motif Containing 31 | 1.139968634 |
| MIR1827 | MicroRNA 1827 | 1.139968634 |
| PDGFRB | Platelet Derived Growth Factor Receptor Beta | 1.109928846 |
| TLR3 | Toll Like Receptor 3 | 1.109928846 |
| NRAS | NRAS Proto-Oncogene, GTPase | 1.109928846 |
| PLAT | Plasminogen Activator, Tissue Type | 1.109928846 |
| ROCK1 | Rho Associated Coiled-Coil Containing Protein Kinase 1 | 1.109928846 |
| CASP10 | Caspase 10 | 1.109928846 |
| PAK4 | P21 (RAC1) Activated Kinase 4 | 1.109928846 |
| VEGFA | Vascular Endothelial Growth Factor A | 1.109928846 |
| PIN1 | Peptidylprolyl Cis/Trans Isomerase, NIMA-Interacting 1 | 1.109928846 |
| YWHAZ | Tyrosine 3-Monooxygenase/Tryptophan 5-Monooxygenase Activation Protein Zeta | 1.109928846 |
| TWIST1 | Twist Family BHLH Transcription Factor 1 | 1.109928846 |
| UBE2C | Ubiquitin Conjugating Enzyme E2 C | 1.109928846 |
| BMP6 | Bone Morphogenetic Protein 6 | 1.109928846 |
| ELK1 | ETS Transcription Factor ELK1 | 1.109928846 |
| PRDX4 | Peroxiredoxin 4 | 1.109928846 |
| BNIP3 | BCL2 Interacting Protein 3 | 1.109928846 |
| BNIP3L | BCL2 Interacting Protein 3 Like | 1.109928846 |
| KDM3A | Lysine Demethylase 3A | 1.109928846 |
| LMO3 | LIM Domain Only 3 | 1.109928846 |
| ZNF32 | Zinc Finger Protein 32 | 1.109928846 |
| MIR200B | MicroRNA 200b | 1.109928846 |
| MIR525 | MicroRNA 525 | 1.109928846 |
| MIR363 | MicroRNA 363 | 1.109928846 |
| TUBB3 | Tubulin Beta 3 Class III | 1.096040606 |
| HSP90B1 | Heat Shock Protein 90 Beta Family Member 1 | 1.096040606 |
| PTPN11 | Protein Tyrosine Phosphatase Non-Receptor Type 11 | 1.075832009 |
| SLC2A1 | Solute Carrier Family 2 Member 1 | 1.075832009 |
| HMOX1 | Heme Oxygenase 1 | 1.075832009 |
| PRKACA | Protein Kinase CAMP-Activated Catalytic Subunit Alpha | 1.075832009 |
| PAK3 | P21 (RAC1) Activated Kinase 3 | 1.075832009 |
| PIK3R2 | Phosphoinositide-3-Kinase Regulatory Subunit 2 | 1.075832009 |
| PPP2CA | Protein Phosphatase 2 Catalytic Subunit Alpha | 1.075832009 |
| CASP6 | Caspase 6 | 1.075832009 |
| CD36 | CD36 Molecule | 1.075832009 |
| CDH3 | Cadherin 3 | 1.075832009 |
| LRP1 | LDL Receptor Related Protein 1 | 1.075832009 |
| PTK6 | Protein Tyrosine Kinase 6 | 1.075832009 |
| GLO1 | Glyoxalase I | 1.075832009 |
| LPAR1 | Lysophosphatidic Acid Receptor 1 | 1.075832009 |
| PAK2 | P21 (RAC1) Activated Kinase 2 | 1.075832009 |
| ADCY10 | Adenylate Cyclase 10 | 1.075832009 |
| EEF2K | Eukaryotic Elongation Factor 2 Kinase | 1.075832009 |
| CEACAM1 | CEA Cell Adhesion Molecule 1 | 1.075832009 |
| GDF2 | Growth Differentiation Factor 2 | 1.075832009 |
| IL17A | Interleukin 17A | 1.075832009 |
| RBL2 | RB Transcriptional Corepressor Like 2 | 1.075832009 |
| SIRPA | Signal Regulatory Protein Alpha | 1.075832009 |
| TRAF2 | TNF Receptor Associated Factor 2 | 1.075832009 |
| MNX1 | Motor Neuron And Pancreas Homeobox 1 | 1.075832009 |
| TNFRSF12A | TNF Receptor Superfamily Member 12A | 1.075832009 |
| VPS37A | VPS37A Subunit Of ESCRT-I | 1.075832009 |
| BAG1 | BAG Cochaperone 1 | 1.075832009 |
| APOBEC3G | Apolipoprotein B MRNA Editing Enzyme Catalytic Subunit 3G | 1.075832009 |
| COL13A1 | Collagen Type XIII Alpha 1 Chain | 1.075832009 |
| RAD9A | RAD9 Checkpoint Clamp Component A | 1.075832009 |
| IFI27 | Interferon Alpha Inducible Protein 27 | 1.075832009 |
| ITPRIP | Inositol 1,4,5-Trisphosphate Receptor Interacting Protein | 1.075832009 |
| BCL2L15 | BCL2 Like 15 | 1.075832009 |
| SNAI2 | Snail Family Transcriptional Repressor 2 | 1.049229741 |
| GLUD1 | Glutamate Dehydrogenase 1 | 1.03538692 |
| NOTCH3 | Notch Receptor 3 | 1.03538692 |
| PTPN1 | Protein Tyrosine Phosphatase Non-Receptor Type 1 | 1.03538692 |
| FASN | Fatty Acid Synthase | 1.03538692 |
| MYH9 | Myosin Heavy Chain 9 | 1.03538692 |
| RPS6KB1 | Ribosomal Protein S6 Kinase B1 | 1.03538692 |
| SIRT1 | Sirtuin 1 | 1.03538692 |
| TPM1 | Tropomyosin 1 | 1.03538692 |
| PPP2R1A | Protein Phosphatase 2 Scaffold Subunit Aalpha | 1.03538692 |
| COL4A2 | Collagen Type IV Alpha 2 Chain | 1.03538692 |
| CTNND1 | Catenin Delta 1 | 1.03538692 |
| CD151 | CD151 Molecule (Raph Blood Group) | 1.03538692 |
| MMP11 | Matrix Metallopeptidase 11 | 1.03538692 |
| SEMA7A | Semaphorin 7A (John Milton Hagen Blood Group) | 1.03538692 |
| ARHGEF7 | Rho Guanine Nucleotide Exchange Factor 7 | 1.03538692 |
| BST2 | Bone Marrow Stromal Cell Antigen 2 | 1.03538692 |
| PPP2R5A | Protein Phosphatase 2 Regulatory Subunit B'Alpha | 1.03538692 |
| PPP2R2D | Protein Phosphatase 2 Regulatory Subunit Bdelta | 1.03538692 |
| CCN1 | Cellular Communication Network Factor 1 | 1.03538692 |
| CCDC178 | Coiled-Coil Domain Containing 178 | 1.03538692 |
| MIR10A | MicroRNA 10a | 1.03538692 |
| MIR30C1 | MicroRNA 30c-1 | 1.03538692 |
| MIR30B | MicroRNA 30b | 1.03538692 |
| SHC1 | SHC Adaptor Protein 1 | 1.013910055 |
| BUB1 | BUB1 Mitotic Checkpoint Serine/Threonine Kinase | 0.982677937 |
| CDC25C | Cell Division Cycle 25C | 0.982677937 |
| BUB3 | BUB3 Mitotic Checkpoint Protein | 0.982677937 |
| FER | FER Tyrosine Kinase | 0.982677937 |
| ITGB5 | Integrin Subunit Beta 5 | 0.982677937 |
| SETD2 | SET Domain Containing 2, Histone Lysine Methyltransferase | 0.982677937 |
| TP73 | Tumor Protein P73 | 0.982677937 |
| BCL2L2 | BCL2 Like 2 | 0.982677937 |
| CDK1 | Cyclin Dependent Kinase 1 | 0.982677937 |
| MAD2L1 | Mitotic Arrest Deficient 2 Like 1 | 0.982677937 |
| SLCO1B3 | Solute Carrier Organic Anion Transporter Family Member 1B3 | 0.982677937 |
| DLG1 | Discs Large MAGUK Scaffold Protein 1 | 0.982677937 |
| EDAR | Ectodysplasin A Receptor | 0.982677937 |
| TDGF1 | Teratocarcinoma-Derived Growth Factor 1 | 0.982677937 |
| PDCD6IP | Programmed Cell Death 6 Interacting Protein | 0.982677937 |
| SCRIB | Scribble Planar Cell Polarity Protein | 0.982677937 |
| SH3GLB1 | SH3 Domain Containing GRB2 Like, Endophilin B1 | 0.982677937 |
| DYNLL2 | Dynein Light Chain LC8-Type 2 | 0.982677937 |
| TSC2 | TSC Complex Subunit 2 | 0.957935095 |
| BAG4 | BAG Cochaperone 4 | 0.9184196 |
| MAP3K7 | Mitogen-Activated Protein Kinase Kinase Kinase 7 | 0.917490005 |
| F10 | Coagulation Factor X | 0.855427086 |
| F3 | Coagulation Factor III, Tissue Factor | 0.855427086 |
| ADAMTSL1 | ADAMTS Like 1 | 0.855427086 |
| SERPINB1 | Serpin Family B Member 1 | 0.855427086 |
| MIR181A1 | MicroRNA 181a-1 | 0.855427086 |
| MAP3K1 | Mitogen-Activated Protein Kinase Kinase Kinase 1 | 0.836247206 |
| CTBP1 | C-Terminal Binding Protein 1 | 0.836247206 |
| CEACAM4 | CEA Cell Adhesion Molecule 4 | 0.802150488 |
| PXN | Paxillin | 0.789446771 |
| MALAT1 | Metastasis Associated Lung Adenocarcinoma Transcript 1 | 0.785730064 |
| IKBKG | Inhibitor Of Nuclear Factor Kappa B Kinase Regulatory Subunit Gamma | 0.737530112 |
| TFDP1 | Transcription Factor Dp-1 | 0.737530112 |
| CRYBA1 | Crystallin Beta A1 | 0.737530112 |
| SERPINE1 | Serpin Family E Member 1 | 0.728532076 |
| FOXO3 | Forkhead Box O3 | 0.727323055 |
| ACTG1 | Actin Gamma 1 | 0.708996356 |
| ARHGDIA | Rho GDP Dissociation Inhibitor Alpha | 0.708996356 |
| EZR | Ezrin | 0.708996356 |
| SLC39A6 | Solute Carrier Family 39 Member 6 | 0.708996356 |
| BIN1 | Bridging Integrator 1 | 0.69874388 |
| TIAM1 | TIAM Rac1 Associated GEF 1 | 0.69874388 |
| PDPK1 | 3-Phosphoinositide Dependent Protein Kinase 1 | 0.694435358 |
| SMAD7 | SMAD Family Member 7 | 0.671585798 |
| NTRK3 | Neurotrophic Receptor Tyrosine Kinase 3 | 0.641545892 |
| RHOC | Ras Homolog Family Member C | 0.641545892 |
| CASP2 | Caspase 2 | 0.633601308 |
| TNC | Tenascin C | 0.610355735 |
| IRF6 | Interferon Regulatory Factor 6 | 0.610355735 |
| HOTAIR | HOX Transcript Antisense RNA | 0.607449174 |
| GNE | Glucosamine (UDP-N-Acetyl)-2-Epimerase/N-Acetylmannosamine Kinase | 0.585381269 |
| XAF1 | XIAP Associated Factor 1 | 0.585381269 |
| SFRP1 | Secreted Frizzled Related Protein 1 | 0.581745505 |
| MAP2K2 | Mitogen-Activated Protein Kinase Kinase 2 | 0.567004085 |
| CSK | C-Terminal Src Kinase | 0.567004085 |
| PIK3C2B | Phosphatidylinositol-4-Phosphate 3-Kinase Catalytic Subunit Type 2 Beta | 0.567004085 |
| FOXC2 | Forkhead Box C2 | 0.567004085 |
| TAGLN | Transgelin | 0.567004085 |
| ARHGDIB | Rho GDP Dissociation Inhibitor Beta | 0.567004085 |
| ENDOG | Endonuclease G | 0.567004085 |
| RACK1 | Receptor For Activated C Kinase 1 | 0.567004085 |
| FBLIM1 | Filamin Binding LIM Protein 1 | 0.567004085 |
| CCDC80 | Coiled-Coil Domain Containing 80 | 0.567004085 |
| PRKD1 | Protein Kinase D1 | 0.558223128 |
| LDHA | Lactate Dehydrogenase A | 0.528183281 |
| ANXA2 | Annexin A2 | 0.528183281 |
| SPP1 | Secreted Phosphoprotein 1 | 0.528183281 |
| SMARCE1 | SWI/SNF Related, Matrix Associated, Actin Dependent Regulator Of Chromatin, Subfamily E, Member 1 | 0.528183281 |
| QSOX1 | Quiescin Sulfhydryl Oxidase 1 | 0.528183281 |
| RBFOX2 | RNA Binding Fox-1 Homolog 2 | 0.528183281 |
