# Supplementary material for: Development and validation of multi-omic prognostic signature of anoikis-related genes in liver hepatocellular carcinoma
Source: Medicine (Baltimore). 2023 Nov 17;102(46):e36190. doi: 10.1097/MD.0000000000036190 (PMC10659623; doi:10.1097/MD.0000000000036190)
Supplement: Supplementary file 2 [file medi-102-e36190-s002.docx]

Supplementary table 2. The primer sequences for qRT-PCR in the current study.

| Gene | Primer sequences |
| --- | --- |
| GAPDH | Forward: ACAACTTTGGTATCGTGGAAGG  Reverse: GCCATCACGCCACAGTTTC |
| SPP1 | Forward: GAAGTTTCGCAGACCTGACAT  Reverse: GTATGCACCATTCAACTCCTCG |
| PLK1 | Forward: CACCAGCACGTCGTAGGATTC  Reverse: CCGTAGGTAGTATCGGGCCTC |
| ETV4 | Forward: GATGAAAGCCGGATACTTGGAC  Reverse: TTCGCGCAAGCTCCCATTT |
| EZH2 | Forward: AATCAGAGTACATGCGACTGAGA  Reverse: GCTGTATCCTTCGCTGTTTCC |
| NQO1 | Forward: GAAGAGCACTGATCGTACTGGC  Reverse: GGATACTGAAAGTTCGCAGGG |
| BSG | Forward: GAAGTCGTCAGAACACATCAACG  Reverse: TTCCGGCGCTTCTCGTAGA |
| PBK | Forward: CCAAACATTGTTGGTTATCGTGC  Reverse: GGCTGGCTTTATATCGTTCTTCT |
